# Establishment of Reference Intervals for Lithium Heparin Plasma Potassium in Adults

**DOI:** 10.3390/diagnostics16213603

**Published:** 2026-11-01

**Authors:** Jingzhao Mei, Xiaoyuan Wang, Wei Huang, Jinyao Ma, Ze An, Jie Feng, Yanhong Gao

**Affiliations:** 1 Department of Clinical Laboratory, The First Medical Center of Chinese PLA General Hospital, Beijing 100853, China; 2 Health Examination Center, The First Medical Center of Chinese PLA General Hospital, Beijing 100853, China

**Keywords:** lithium heparin plasma, potassium, biochemical testing, difference analysis, reference intervals

## Abstract

**Background/Objectives**: In current Chinese clinical practice, the reference interval established for serum potassium is routinely applied to both routine biochemical testing conducted with serum separator tubes and emergency biochemical testing using lithium heparin plasma. This study aimed to establish a plasma-specific reference interval for potassium via the indirect ion-selective electrode (ISE) method, in compliance with the Chinese national health industry standard WS/T 402-2024. **Methods**: A total of 296 paired plasma and serum samples were collected from health examination participants at the Chinese PLA General Hospital. Potassium concentrations were measured using the ISE module of the Roche cobas 8000 modular analyzer series. The Wilcoxon signed-rank test and Bland–Altman analysis were used to compare the two specimen types, and reference intervals for plasma potassium were determined using the nonparametric method. **Results**: Serum potassium concentrations were significantly higher than plasma potassium levels (median difference: 0.23 mmol/L; *Z* = −13.96; *p* < 0.001). Statistical analysis indicated that plasma potassium reference intervals should be stratified by sex. The reference interval for plasma potassium was 3.21–4.72 mmol/L in adult males and 3.21–4.51 mmol/L in adult females. **Conclusions**: Plasma and serum potassium levels exhibit a systematic bias. Retrospective analysis shows that applying the serum-derived reference interval to plasma potassium results in reclassification of 16.97% of emergency plasma potassium measurements. The sex-specific plasma potassium reference intervals established in this study offer a more reliable basis for interpreting plasma potassium results in emergency and perioperative settings.

## 1. Introduction

Potassium is a key electrolyte essential for maintaining human body homeostasis [1] and the most abundant intracellular cation. Total body potassium in healthy adults is approximately 3500–4500 mmol, with about 98% residing intracellularly and approximately 2% in the extracellular compartment. The body exhibits a characteristic ion distribution pattern of high extracellular sodium and high intracellular potassium: the extracellular sodium concentration ranges from 140 to 150 mmol/L, whereas the extracellular potassium concentration is only 3.5–5.5 mmol/L. This transmembrane ion concentration gradient is primarily maintained by active transport via the Na^+^/K^+^-ATPase (sodium–potassium pump) and forms the fundamental basis for nerve conduction, myocardial contraction, and skeletal muscle function [2,3]. Given its decisive role in determining cell membrane potential, even minor deviations in serum potassium concentration can have serious clinical consequences. Epidemiological evidence has demonstrated a U-shaped association between blood potassium levels and all-cause as well as cardiovascular mortality, with the lowest risk observed at approximately 4.2 mmol/L; hyperkalemia can significantly increase mortality risk in patients with cardiovascular disease [4]. Therefore, accurate measurement of serum potassium is a critical basis for clinical decision-making in emergency, perioperative, and critical care settings [5].

The emergency department of our hospital handles a large volume of surgical cases, and the emergency biochemical test results that clinicians rely on to assess patients’ potassium status are predominantly derived from lithium heparin plasma specimens. The advantages of using lithium heparin plasma are twofold: first, it enables rapid plasma separation and avoids interference from potassium release from platelets and erythrocytes during the clotting process, thereby producing potassium measurements that more closely reflect in vivo levels; second, it effectively reduces laboratory turnaround time, meeting the demand for rapid screening in emergency settings [6].

Multiple studies have confirmed that serum potassium concentrations are significantly higher than plasma potassium concentrations, a difference that is primarily attributable to potassium release from platelets and blood cells during the clotting process [7,8,9]. However, in current Chinese clinical practice, the reference interval established for serum potassium is universally applied both to serum specimens collected in serum separator tubes (for routine biochemical testing) and lithium heparin plasma specimens (for emergency biochemical testing). This practice poses a clear clinical risk: evaluating plasma potassium results against the serum-derived reference interval may lead to underestimation of patients’ potassium status, thereby misleading clinical decision-making, potentially delaying emergency surgical interventions, and adversely affecting patient outcomes. Therefore, it is necessary to establish a dedicated reference interval for plasma potassium.

Potassium was measured using an indirect ion-selective electrode (ISE) on the ISE module of the Roche cobas 8000 modular analyzer series. The manufacturer’s instructions for use (IFU) for this assay provide separate reference intervals for serum and lithium heparin plasma potassium derived from Western populations (3.5–5.1 mmol/L and 3.4–4.5 mmol/L, respectively), both cited from Tietz Fundamentals of Clinical Chemistry, 5th edition (2001) [10]. In China, the current national industry standard WS/T 404.3-2012 recommends a serum potassium reference interval of 3.5–5.3 mmol/L [11]; a study by Jia et al. involving 4524 healthy Han adults reported an interval of 3.6–5.2 mmol/L [12]; and our laboratory has historically adopted an interval of 3.5–5.5 mmol/L [13] sourced from domestic textbooks, which is also specific to serum potassium. None of these sources specify reference limits for lithium heparin plasma potassium.

At present, there are no national industry standards for plasma potassium reference intervals in China. In accordance with the Chinese national health industry standard WS/T 402-2024 [14], this study aimed to establish a reference interval for potassium in lithium heparin plasma using the ion-selective electrode method, with the goal of providing a more accurate interpretive basis for emergency and clinical biochemical testing.

## 2. Materials and Methods

### 2.1. Study Population

In accordance with the Chinese national health industry standard WS/T 402-2024 and the EP28-A3c [15] guideline, adults who underwent health examinations at the Health Examination Center of our hospital were selected as reference individuals. A total of 416 reference individuals aged 18–84 years were initially enrolled, comprising 215 males and 201 females, with a relatively even distribution across 10-year age intervals. Inclusion criteria were as follows: (1) no recent diagnosis of disease; (2) no acute or chronic infections, hematological disorders, cardiovascular and cerebrovascular diseases, metabolic diseases, or malignancies; (3) no surgical history within the preceding 6 months and no medication use within the preceding 2 weeks; (4) no recent history of blood donation or transfusion; and (5) for females, not currently pregnant or lactating. Individuals with abnormal routine biochemical test results (including liver and kidney function, homocysteine, and thyroid function), as well as samples with visible hemolysis, icterus, or lipemia, were excluded. Ultimately, a total of 296 subjects (148 males and 148 females) were included to establish the reference intervals.

### 2.2. Instruments, Consumables, and Detection Principle

Potassium measurements were performed on the ISE module of the cobas 8000 modular analyzer series (Roche Diagnostics, Rotkreuz, Switzerland), used in conjunction with the cobas p 671 medical centrifuge and the cobas p 701 post-processing system. The blood collection tubes used were 5 mL serum separator vacuum tubes (13 × 100 mm) and 3 mL lithium heparin vacuum tubes (13 × 75 mm) (Greiner Bio-One GmbH, Kremsmünster, Austria). All lithium heparin tubes used in this study were coated with spray-dried lithium heparin powder on the inner tube wall rather than liquid anticoagulant; therefore, anticoagulant-induced specimen dilution was negligible, and lithium-heparin plasma samples can approximate in vivo potassium concentrations. Potassium ion (K^+^) concentrations were determined using the indirect ion-selective electrode (ISE) method.

### 2.3. Experimental Procedures

Prior to the study, the biochemical analyzer underwent routine maintenance and calibration. Routine internal quality control was performed using matched high- and low-level control materials, and all quality control results during the study period fell within the acceptable ranges defined by the Westgard rules. The precision profile of the potassium assay was verified in our laboratory in accordance with industry standard WS/T 408-2024 [16] at two concentration levels: Level 1 (mean = 3.526 mmol/L): within-run CV = 0.25%, within-laboratory total CV = 1.73%; Level 2 (mean = 6.051 mmol/L): within-run CV = 0.23%, within-laboratory total CV = 0.29%. Study samples were analysed in multiple batches, with calibration and internal quality control conducted for each batch to ensure consistent analytical performance.

Fasting venous blood was collected from all 416 reference individuals into both lithium heparin tubes and serum separator tubes simultaneously. Within 2 h of collection, samples were centrifuged at 2300× *g* for 7 min to separate plasma and serum, followed by immediate potassium measurement on the analyzer. Statistical analysis was performed in a fully anonymized manner. This study was approved by the Ethics Committee of Chinese PLA General Hospital (Approval No. S2024-596-01).

### 2.4. Statistical Analysis and Reference Interval Establishment

Statistical analyses were performed using SPSS 27.0. Paired comparisons of plasma and serum potassium concentrations from the same individual were conducted. As the differences were non-normally distributed (Shapiro–Wilk test: *W* = 0.92, *p* < 0.001), the Wilcoxon signed-rank test was applied for these paired comparisons. Non-normally distributed data were expressed as median (interquartile range) [M (P25–P75)], with *p* < 0.05 considered statistically significant. The significance level was set at α = 0.05. Spearman rank correlation analysis was used to assess the correlation between the two specimen types, with *r* > 0.75 indicating a relevant correlation. The Bland–Altman method was employed to evaluate agreement between the two specimen types, calculating the mean bias and 95% limits of agreement (LoA).

Reference intervals were established following the standard procedure outlined in WS/T 402-2024: (1) scatter plots of concentration versus age were constructed to visually assess differences between sexes and age groups and identify extreme outliers; (2) the standard normal deviate test was used to evaluate sex differences and determine whether partitioning was necessary; (3) Dixon’s method was applied to identify and handle outliers; (4) the nonparametric percentile method was used to determine reference intervals, with the 2.5th percentile as the lower limit and the 97.5th percentile as the upper limit; and (5) the bootstrap method was used to calculate 90% confidence intervals for the reference limits.

## 3. Results

### 3.1. Comparison and Bias Analysis of Potassium Between Plasma and Serum Specimens

**Figure 1:**
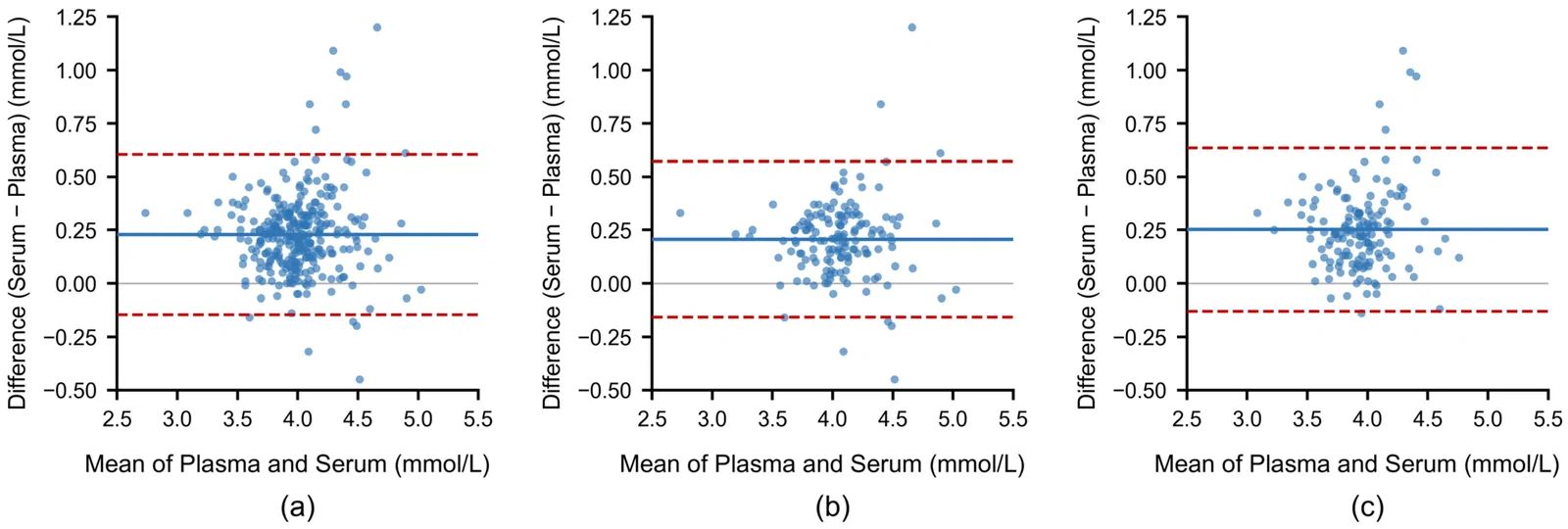
Bland–Altman plots comparing serum and plasma potassium. Difference is defined as serum potassium minus plasma potassium. Blue solid lines indicate the mean bias; red dashed lines indicate the 95% limits of agreement (LoA). (**a**) Overall cohort (*n* = 296): mean bias = +0.23 mmol/L, SD = 0.19, 95% LoA = −0.15–+0.61. (**b**) Males (*n* = 148): mean bias = +0.21 mmol/L, SD = 0.19, 95% LoA = −0.16–+0.57. (**c**) Females (*n* = 148): mean bias = +0.25 mmol/L, SD = 0.20, 95% LoA = −0.13–+0.63.

Potassium concentrations in matched plasma and serum specimens from the same individuals were measured using the analyzer. As shown in Table 1, serum potassium was significantly higher than plasma potassium, with a statistically significant difference (*p* < 0.01). Further agreement analysis using the Bland–Altman method is illustrated in Figure 1. The median difference between serum and plasma potassium was 0.23 mmol/L (95% CI: 0.20–0.25 mmol/L), with 95% limits of agreement ranging from −0.15–0.61 mmol/L. As the 95% CI of the mean difference did not include zero, a systematic bias existed between the two specimen types, indicating they are not interchangeable. Sex-related differences were observed in both matrices, and potassium concentrations were consistently higher in males than in females. The mean plasma potassium was 3.96 mmol/L in males and 3.82 mmol/L in females (mean difference: 0.14 mmol/L; median difference: 0.13 mmol/L; *p* < 0.001). The mean serum potassium was 4.17 mmol/L in males and 4.07 mmol/L in females (mean difference: 0.09 mmol/L; median difference: 0.11 mmol/L; *p* = 0.002). Detailed data are provided in Supplementary Table S1.

Further bias analysis was performed on the plasma and serum data. According to the allowable bias (B%) specified in the Chinese national health industry standard (WS/T 403-2024) and our laboratory’s quality requirements, the bias for potassium exceeded the allowable range, as shown in Table 2. Serum potassium concentrations were consistently higher than plasma potassium concentrations.

### 3.2. Establishment of Reference Intervals for Plasma Potassium

**Figure 2:**
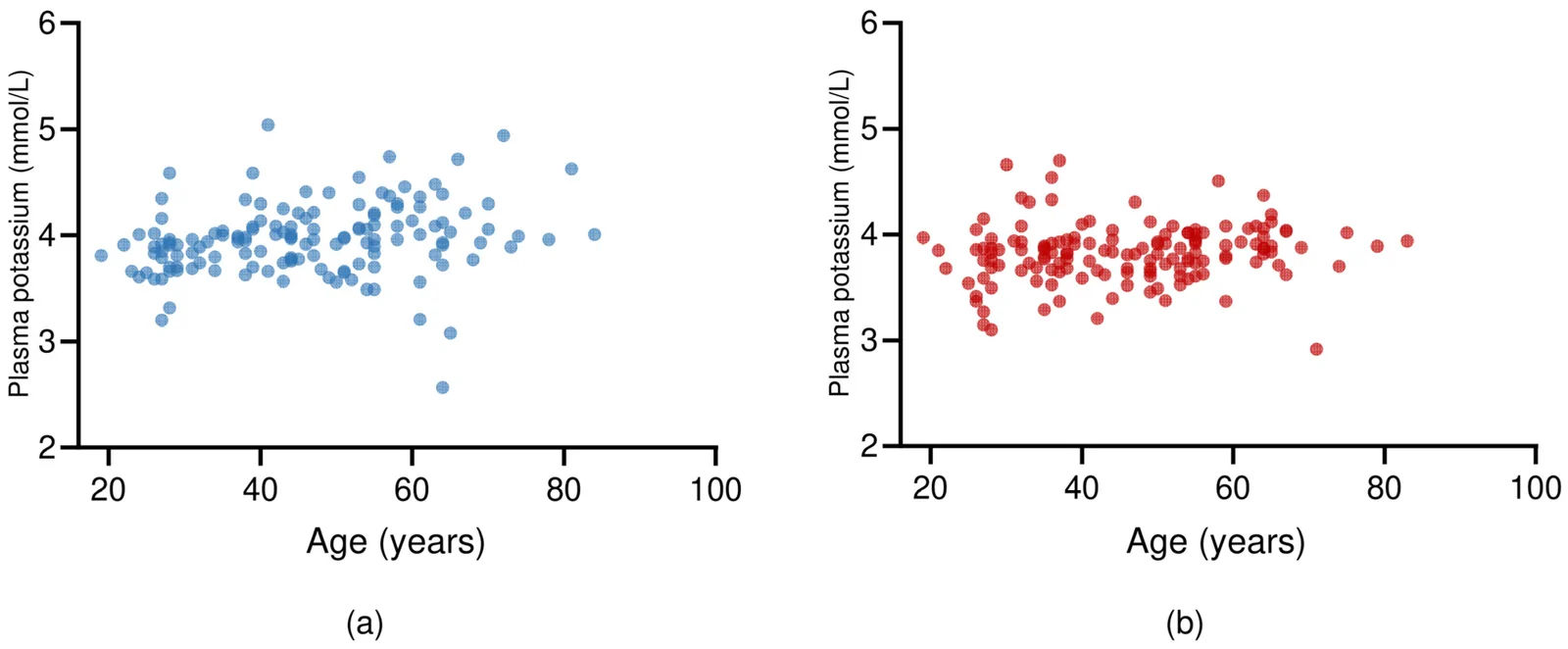
Scatter plot of plasma potassium reference values stratified by age. (**a**) Males; (**b**) females. Plasma potassium showed a weak positive correlation with age (Spearman’s *ρ* = 0.223, *p* < 0.001). Regression fitting results are provided in Supplementary Figure S1.

Scatter plots of the measured reference values are shown in Figure 2. Plasma potassium was weakly positively associated with age (Spearman’s *ρ* = 0.223, *p* < 0.001), increasing by +0.038 mmol/L per decade (95% CI: 0.013–0.063). The association was stronger in males (*ρ* = 0.285, *p* < 0.001; +0.050 mmol/L per decade) and weaker in females (*ρ* = 0.186, *p* = 0.024). The regression slope for females was non-significant (+0.023 mmol/L per decade, 95% CI: −0.009–0.055; *p* = 0.16). Harris–Boyd testing (*z* = 1.85 and 0.76 vs. z* = 2.36) did not support age partitioning of reference intervals (Supplementary Figure S1, Supplementary Table S2). Next, the standard normal deviate test (*Z* test) was applied to evaluate sex differences and determine whether partitioning was necessary. *z* = $\frac{{\bar{x}}_{1}-{\bar{x}}_{2}}{\sqrt{\frac{s_{1}^{2}}{n_{1}}+\frac{s_{2}^{2}}{n_{2}}}}$3.94, z* = $3\sqrt{\frac{n_{average}}{120}}=3\sqrt{\frac{n_{1}\;+\;n_{2}}{240}}$ = 3.33. Since *z* > z*, partitioning by sex was deemed appropriate. Outliers were then identified by visual inspection and Dixon’s method. Measured values were sorted in ascending order, and the range *R* (difference between the maximum and minimum values) was calculated. The difference *D* between each extreme value and its nearest neighbor was then computed; if *D*/*R* ≥ 1/3, the extreme observation was considered an outlier. For males, *R* = 2.47, with the two *D* values being 0.51 and 0.10; for females, *R* = 1.78, with the two *D* values being 0.18 and 0.04. All *D*/*R* ratios were less than 1/3, indicating no outliers in either group. In accordance with the recommendation of the Chinese national health industry standard WS/T 402-2024, the nonparametric method was used to determine the reference intervals. The lower and upper reference limits were derived from the 2.5th and 97.5th percentiles, respectively. The 90% confidence intervals for the reference limits were calculated using the bootstrap method. The established reference intervals are summarized in Table 3.

### 3.3. Impact of the Newly Established Reference Intervals on the Classification of Emergency Plasma Potassium Results

To further evaluate the clinical utility of the newly established reference intervals and assess how routinely reported results would shift if plasma-specific intervals were adopted, we retrospectively analyzed 36,555 lithium heparin plasma potassium measurements from emergency biochemistry testing at our hospital over a 38-day period (20,864 males, 15,691 females). All results were reclassified using the sex-specific plasma reference intervals (males: 3.21–4.72 mmol/L; females: 3.21–4.51 mmol/L) instead of the currently adopted serum-based interval (3.5–5.5 mmol/L).

Because the lower limits of the plasma intervals were identical in both sexes, the lower discordance zone was shared: 3960 measurements (10.83% of all results) with potassium concentrations of 3.21–3.50 mmol/L were classified as hypokalemia under the serum interval but fell within the reference range under the new plasma intervals. The upper discordance zone was sex-specific: 1062 of 20,864 measurements in males (5.09%) at 4.72–5.50 mmol/L and 1182 of 15,691 measurements in females (7.53%) at 4.51–5.50 mmol/L, totaling 2244 measurements (6.14% of all results), were within the serum reference range but would be classified as hyperkalemia under the new plasma intervals. Overall, 16.97% of emergency plasma potassium results would be reclassified if the newly established intervals were applied (Table 4).

Since there is no independent reference standard for an individual patient’s true potassium status, this analysis was designed to describe shifts in existing reported classifications rather than to determine which classification is more accurate.

## 4. Discussion

Potassium, as the most abundant cation in intracellular fluid, plays an essential role in maintaining cell membrane potential, regulating neuromuscular excitability, and sustaining cardiac electrophysiological function [17]. Accurate measurement of blood potassium concentrations is highly significant for clinical diagnostic and therapeutic decision-making, particularly in preoperative assessment for emergency surgery. The findings of this study demonstrate a significant difference between lithium heparin plasma potassium and serum potassium, with serum potassium concentrations markedly higher than plasma potassium concentrations. Moreover, this difference exceeded the clinically acceptable range, indicating that the current serum potassium reference interval is not applicable to interpreting plasma potassium results. In accordance with the Chinese national health industry standard WS/T 402-2024, this study established, for the first time, reference intervals for lithium heparin plasma potassium using the ion-selective electrode method in a healthy examination population at the Chinese PLA General Hospital. The results showed that when stratified by sex, the reference interval for plasma potassium was 3.21–4.72 mmol/L in males and 3.21–4.51 mmol/L in females.

### 4.1. Mechanistic Interpretation and Quantitative Evaluation of Serum–Plasma Potassium Differences

It is known that the difference between serum and plasma potassium arises mainly from in vitro events during blood clotting [18]. During serum preparation, blood must stand at room temperature for 30–60 min to allow complete clotting. During this period, platelets become activated and rupture, releasing intracellular potassium ions; erythrocytes may also continuously release small amounts of potassium via ongoing metabolism, ultimately resulting in higher potassium concentrations in serum than in plasma [19,20]. In contrast, lithium heparin plasma can be centrifuged and separated within minutes after blood collection, which helps to minimize the impact of ex vivo cellular metabolism on potassium concentrations and provides a closer reflection of in vivo potassium levels. Paired sample analysis in this study showed that serum potassium concentrations were significantly higher than plasma potassium concentrations (median difference: 0.23 mmol/L; *Z* = −13.96; *p* < 0.001). Bland–Altman agreement analysis further revealed a systematic bias between the two specimen types (mean bias = 0.23 mmol/L; 95% limits of agreement: −0.15 to 0.61 mmol/L),which exceeded the allowable bias criteria for potassium based on within-subject biological variation [21]. Collectively, these quantitative findings demonstrate that serum potassium reference intervals are unsuitable for interpreting lithium-heparin plasma potassium results, reinforcing the need for matrix-specific reference intervals for plasma potassium.

### 4.2. Sex Differences in Plasma Potassium Reference Intervals

In this study, the standard normal deviate test (*Z* test) was applied to analyze sex differences in plasma potassium reference intervals, yielding statistically significant differences between males and females (*p* < 0.05). According to the Chinese national health industry standard WS/T 402-2024, when sex differences in reference intervals are present but their clinical significance remains uncertain, a thorough evaluation incorporating clinical input should be conducted. This study contends that sex-specific reference intervals are nonetheless warranted for two reasons: first, in emergency surgery settings, potassium level assessment directly informs surgical decision-making, and the lower upper reference limit in females enables more sensitive identification of hyperkalemia risk; second, modern laboratory information systems can automatically match sex-appropriate reference intervals, presenting no technical barriers to clinical implementation.

This sex difference may be attributable to the regulatory effects of sex hormones on potassium homeostasis and the difference in total body potassium content driven by sex-based variations in muscle mass [22,23]. Notably, this difference is not confined to lithium heparin plasma: serum potassium shows a sex difference of similar direction and magnitude, indicating that the phenomenon reflects an intrinsic biological difference rather than an anticoagulant-induced matrix effect. It is also necessary to distinguish statistical significance from the criteria used for partitioning. Although the sex difference in serum potassium was statistically significant (*p* = 0.002), it did not meet the partitioning threshold specified in WS/T 402-2024 (*Z* = 2.55 vs. Z* = 3.33), whereas the between-sex difference in plasma potassium satisfied the partitioning criterion (*Z* = 3.94). Whether serum potassium requires sex-specific partitioning is beyond the scope of this study.

### 4.3. Comparison with Manufacturer and Literature Intervals

The central tendency of plasma potassium in our cohort agreed closely with the plasma reference interval stated in the manufacturer’s package insert (3.4–4.5 mmol/L; midpoint, 3.95 mmol/L), with median values of 3.96 mmol/L for males and 3.83 mmol/L for females. However, the sex-specific intervals derived in this study were notably wider: 3.21–4.72 mmol/L for males (span, 1.51 mmol/L) and 3.21–4.51 mmol/L for females (span, 1.30 mmol/L), compared with a span of only 1.10 mmol/L for the manufacturer’s interval, which does not stratify by sex.

Several factors may account for this discrepancy. First, the manufacturer’s interval is derived from historical 2001 data, with limited descriptions of the source population and pre-analytical procedures, and no sex stratification. Second, in our cohort, potassium values were more widely dispersed in men than in women (SD 0.33 vs. 0.28 mmol/L), so a single pooled interval cannot accommodate both distributions; the wider limits observed in males are thus more consistent with sex-specific biological variation than with random error. Third, with 148 participants per sex, the reference limits themselves carry appreciable uncertainty. Bootstrap estimation of the 90% confidence intervals for these limits yielded ranges of 3.08–3.56 mmol/L (lower) and 4.55–4.94 mmol/L (upper) for males, and 3.10–3.37 mmol/L and 4.31–4.66 mmol/L for females. The manufacturer’s lower limit of 3.4 mmol/L fell within the confidence interval of the male lower limit, while its upper limit of 4.5 mmol/L fell within that of the female upper limit; only the male upper limit and female lower limit differed from the manufacturer’s values beyond this range of uncertainty.

From a clinical standpoint, a more relevant comparator than the manufacturer’s interval is the serum reference interval previously used in our laboratory (3.5–5.5 mmol/L). That interval is wider (span, 2.00 mmol/L) and lies 0.4–0.5 mmol/L above the plasma intervals established in this study. This systematic offset is precisely what drives the reclassification of plasma potassium results when serum intervals are applied. Relative to the previous serum interval, the plasma-specific intervals established in this study are narrower and provide better discriminatory performance.

Jia et al. established a serum potassium reference interval of 3.6–5.2 mmol/L in 4524 Chinese Han adults using indirect ion-selective electrode measurement, with no sex stratification. Applying the mean serum-to-plasma difference of 0.23 mmol/L reported in that study yields an equivalent plasma interval of approximately 3.37–4.97 mmol/L. The upper limit of this derived interval agrees well with the sex-specific plasma intervals established in this study, whereas the lower limit is higher. This suggests that even a large-sample serum reference interval from a Chinese population cannot be directly extrapolated to lithium heparin plasma samples.

### 4.4. Retrospective Analysis in the Emergency Setting and Its Clinical Implications

To further evaluate the clinical applicability of the newly established reference intervals, this study retrospectively analyzed a total of 36,555 lithium heparin plasma potassium results from emergency biochemical testing at our hospital over 38 days. The results demonstrated that when the original serum potassium reference interval (3.5–5.5 mmol/L) was applied, 16.97% of emergency plasma potassium results were reclassified.

This finding indicates that applying the serum potassium reference interval to evaluate emergency plasma potassium can lead to misdiagnosis in approximately one-sixth of emergency patients. In the context of emergency surgery, a diagnosis of pseudohypokalemia may trigger unnecessary intravenous potassium supplementation, which not only increases the risk of iatrogenic hyperkalemia but may also result in the postponement of non-urgent surgical procedures, thereby compromising timely patient treatment [24]. Multiple studies have demonstrated that preoperative hypokalemia is significantly associated with an increased risk of postoperative arrhythmias, prolonged hospital stay, and other complications [25,26]. Therefore, establishing dedicated reference intervals for plasma potassium is of substantial practical significance for safeguarding patient safety in emergency and perioperative settings.

The present analysis carries inherent limitations. The emergency-department dataset was retrospective and lacked an independent potassium baseline to serve as a gold standard; clinical diagnoses, medications, and outcomes could not be retrieved in full. The reclassification rate therefore reflects only the extent to which result interpretation would change and should not be equated with a diagnostic error rate. In addition, the unit of analysis was the individual test result rather than the patient, so repeated measurements in the same patient may have influenced the distribution; this confounding may also be related to the sex differences observed within the discordance zones. We report this observation without inferring causality. Further evaluation of the clinical performance of the new intervals would require an independent cohort of healthy individuals, preferably in a multicenter design, or a prospective study in which classification by the new intervals is analyzed against an independent reference standard and clinical outcomes. Finally, indirect methods for reference interval determination [27,28] merit exploration as a complementary approach for large emergency datasets, although the predominance of acutely ill patients in our cohort violates their core assumption that the dataset be enriched with presumably healthy individuals; sampling uncertainty-based verification strategies [28] may facilitate future comparisons between directly and indirectly derived intervals.

### 4.5. Limitations of This Study

This study has several limitations. First, all participants were part of the health examination population at the Chinese PLA General Hospital, so the geographic representativeness of the sample needs further expansion, and future studies with larger sample sizes are required for validation. Additionally, although the nonparametric method used for reference interval establishment complies with the requirements of WS/T 402-2024, differences exist between analytical systems in terms of electrode characteristics, matrix effects, and calibrator traceability [29]. Therefore, before applying the findings of this study, individual laboratories should verify the reference intervals on their local analytical systems and within their local healthy populations, rather than directly adopting the values reported herein.

Collectively, there is a clinically meaningful systematic difference between lithium heparin plasma potassium and serum potassium, and they should not share a common reference interval. Sex-related biological variation should be fully evaluated when establishing plasma potassium reference intervals, with stratification implemented if indicated.

## 5. Conclusions

In conclusion, there is a clinically significant difference between plasma and serum potassium, and the current serum potassium reference interval cannot be used to accurately evaluate patients’ actual plasma potassium levels. The sex-specific plasma potassium reference intervals established in this study, which comply with the Chinese national health industry standard (males: 3.21–4.72 mmol/L; females: 3.21–4.51 mmol/L), can offer a more accurate and scientific basis for interpreting emergency and clinical biochemical tests, and have important value for clinical application.

## Figures and Tables

**Table 1:** Comparison and correlation analysis of potassium concentrations between plasma and serum specimens.

Item	*n*	Plasma Group	Serum Group	Median Difference (95%CI)	*Z*	*p*	Spearman *Rho*
K/(mmol/L), Total	296	3.89 (3.71, 4.04)	4.10 (3.94, 4.29)	0.23 (0.20, 0.25)	−13.96	<0.001	0.81
Males	148	3.96 (3.78, 4.09)	4.16 (3.97, 4.35)	0.21 (0.18, 0.24)	−9.48	<0.001	0.84
Females	148	3.83 (3.68, 3.95)	4.06 (3.90, 4.21)	0.24 (0.21, 0.26)	−10.25	<0.001	0.76

Data are presented as median (P25, P75). Wilcoxon’s signed-rank test was used for paired comparisons, and Spearman’s *ρ* for correlation. The median difference was calculated as serum minus plasma potassium. CI—confidence interval.

**Table 2:** Bias analysis of potassium concentrations between plasma and serum specimens.

Item	*n*	Bias (%)	Allowable Bias (%)
K/(mmol/L) Total	296	5.89	2.00
Males	148	5.30	2.00
Females	148	6.54	2.00

Bias (%) represents the percentage difference between serum and plasma potassium concentrations. Allowable bias refers to the predefined acceptable limit for potassium.

**Table 3:** Reference intervals for plasma potassium in males and females.

Item	*n*	Mean	Median	Reference Interval	90% CI for Lower Limit	90% CI for Upper Limit
K/(mmol/L) Males	148	3.96	3.96	3.21–4.72	[3.08, 3.56]	[4.55, 4.94]
Female	148	3.82	3.83	3.21–4.51	[3.10, 3.37]	[4.31, 4.66]

Reference limits were estimated using the non-parametric method specified in WS/T 402-2024; 90% confidence intervals were derived via bootstrap resampling (B = 10,000).

**Table 4:** Discrepancies in classification between the serum potassium reference interval and the newly established plasma potassium reference intervals.

Potassium Interval (mmol/L)	Serum RI	Plasma RI	Males, *n* (%)	Females, *n* (%)	Total, *n* (%)
<3.21	Hypokalemia	Hypokalemia	1305 (6.25)	270 (1.72)	1575 (4.31)
3.21–3.50	Hypokalemia	Normal	3285 (15.74)	675 (4.30)	3960 (10.83)
3.50–4.72 Males/ 3.50–4.51 Females	Normal	Normal	15,012 (71.95)	13,504 (86.06)	28,516 (78.01)
4.72–5.50 Males/ 4.51–5.50 Females	Normal	Hyperkalemia	1062 (5.09)	1182 (7.53)	2244 (6.14)
>5.50	Hyperkalemia	Hyperkalemia	200 (0.96)	60 (0.38)	260 (0.71)
Total reclassified			4347 (20.83)	1857 (11.83)	6204 (16.97)
Classification unchanged			16,517 (79.17)	13,834 (88.170)	30,351 (83.03)

Percentages are calculated based on sex-specific total tests (20,864 males, 15,691 females) or all 36,555 measurements. Reclassification refers to a change in diagnostic category caused by switching reference intervals. Only two directional category transitions occurred due to the relative positions of serum and plasma cutoffs. Without an independent reference standard for true potassium status, these proportions describe the magnitude of classification shift rather than diagnostic error. The unit of analysis is the test result, not the patient.

## Data Availability

The data presented in this study are available on request from the corresponding authors due to ethical and patient privacy restrictions.

## Supplementary Materials

The following supporting information can be downloaded at https://www.mdpi.com/article/10.3390/diagnostics16213603/s1, Figure S1: Scatter plots of plasma potassium concentration versus age, with fitted ordinary least-squares regression lines; Table S1: Sex difference in potassium concentration in paired plasma and serum samples; Table S2: Spearman correlation and linear regression of plasma potassium concentration versus age, stratified by sex.

## Abbreviations

The following abbreviations are used in this manuscript:

ISE	ion-selective electrode
RI	reference interval
CI	confidence interval
SD	standard deviation
CV	coefficient of variation
LoA	limits of agreement
