# Supplementary material for: Establishment of Reference Intervals for Lithium Heparin Plasma Potassium in Adults
Source: Diagnostics (Basel). 2026 Nov 1;16(21):3603. doi: 10.3390/diagnostics16213603 (PMC13761456; doi:10.3390/diagnostics16213603)
Supplement: Supplementary file 1 [file diagnostics-16-03603-s001.zip › diagnostics-4624005-supplementary.pdf]

Supplementary Information

Establishment of Reference Intervals for Lithium Heparin Plasma Potassium in Adults

Jingzhao Mei[1] †, Xiaoyuan Wang[2] †, Wei Huang[1], Jinyao Ma[1], Ze An[1], Jie Feng[1] * and Yanhong Gao[1] *

[1] Department of Clinical Laboratory,The First Medical Center of Chinese PLA General Hospital, Beijing 100853, China
[2] Health Examination Center, The First Medical Center of Chinese PLA General Hospital, Beijing 100853, China
* Correspondence: Jie Feng, fengjie654321@126.com; Tel: [China-Beijing -01066935345]; Yanhong Gao,E-mail: gyh25cn@126.comTel: [China-Beijing -01066937374]
† These authors contributed equally to this work. .
* These authors are co-corresponding authors.

Supplementary Table S1. Sex difference in potassium concentration in paired plasma and serum samples.

Matrix	Median, mmol/L (males / females)	Mean, mmol/L (males / females)	Median difference (males / females)	Mean difference (males / females)	$P^{\dagger}$	z	z^*	Partition
Plasma potassium	3.96 / 3.83	3.96 / 3.82	+0.13	+0.14	2.0×10^{-5}	3.94	3.33	Yes
Serum potassium	4.16 / 4.06	4.17 / 4.07	+0.11	+0.09	0.002	2.55	3.33	No

† Mann–Whitney *U* test, two-sided, males versus females.

‡ $z = (\bar{x}_1 - \bar{x}_2) / \sqrt{(s_1^2/n_1 + s_2^2/n_2)}$, calculated from means; $z^* = 3\sqrt{[(n_1 + n_2)/240]}$. Separate reference intervals are indicated when z exceeds z^* (WS/T 402–2024).Plasma and serum were obtained from the same individuals: $n = 148$ men and 148 women (296 in total).

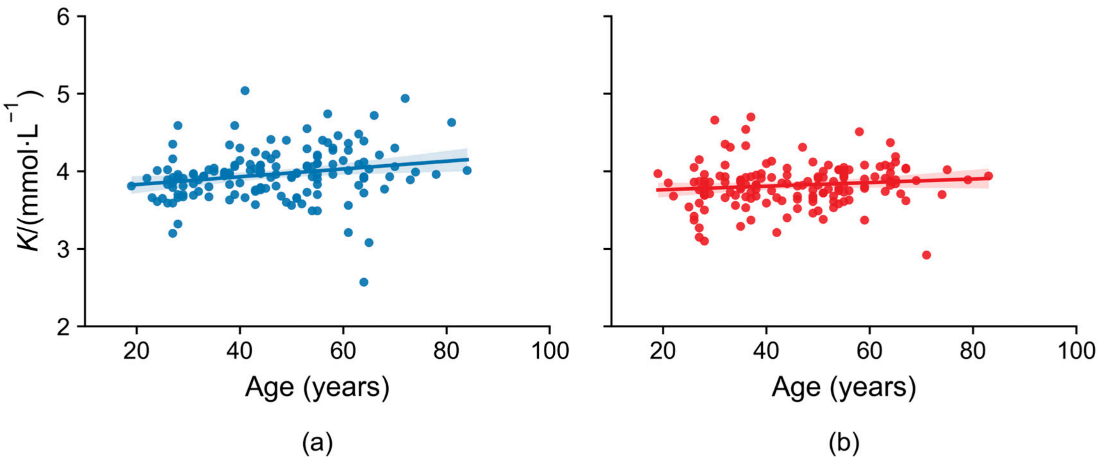

Supplementary Figure S1. Scatter plots of plasma potassium concentration versus age, with fitted ordinary least-squares regression lines. (A) Males; **(B)** females. Shaded areas represent the 95% confidence interval of the fitted mean. The slope was +0.050 mmol/L per 10 years (95% CI, 0.014–0.087; $P = 0.008$) in males and +0.023 mmol/L per 10 years (95% CI, −0.009 to 0.055; $P = 0.157$) in females; for all subjects, the slope was +0.038 mmol/L per 10 years (95% CI, 0.013–0.063; $P = 0.003$), with no significant age × sex interaction ($P = 0.273$). Spearman rank correlations: $\rho = 0.285$ ($P < 0.001$) for males, $\rho = 0.186$ ($P = 0.024$) for females, and $\rho = 0.223$ ($P < 0.001$) for all subjects.

Supplementary Table S2. Spearman correlation and linear regression of plasma potassium concentration versus age, stratified by sex.

**Group**	***n***	**ρ**	***P* (ρ)**	**β, mmol/L per 10 years (95% CI)**	***P***	**R^2**
Males	148	0.285	< 0.001	+0.050 (0.014 to 0.087)	0.008	0.048
Females	148	0.186	0.024	+0.023 (−0.009 to 0.055)	0.157	0.014
All subjects (pooled)	296	0.223	< 0.001	+0.038 (0.013 to 0.063)	0.003	0.030

β, slope of the linear regression of plasma potassium concentration on age, expressed as the change in mmol/L per 10-year increment of age. CI, confidence interval; R2, coefficient of determination. The Harris–Boyd partitioning criterion, with a 45-year cut-off corresponding to the cohort (median age (45 years in males, 46 years in females), yielded $z = 1.85$ in males and $z = 0.76$ in females (n = 73 and 75 per stratum, respectively), calculated from stratum means. Both values are below the critical value $z^* = 3\sqrt{(148/240)} = 2.36$, indicating that age-specific reference intervals are not warranted.
